# Young Woman with Leg Lesions

**DOI:** 10.5811/cpcem.2021.3.51733

**Published:** 2021-04-16

**Authors:** Daniel Finnin

**Affiliations:** Albany Medical Center, Department of Emergency Medicine, Albany, New York

**Keywords:** Pyoderma gangrenosum, case report, ulcerative colitis

## Abstract

**Case Presentation:**

The patient was a 33-year-old woman with inflammatory bowel disease presenting for worsening lower leg lesions with significant pain recalcitrant to oral doxycycline.

**Discussion:**

Pyoderma gangrenosum is a rare ulcerative skin condition with significant pain that is often associated with other systemic diseases typically treated with immunosuppressive medications aimed at the underlying cause.

## CASE PRESENTATION

**Image 1:**
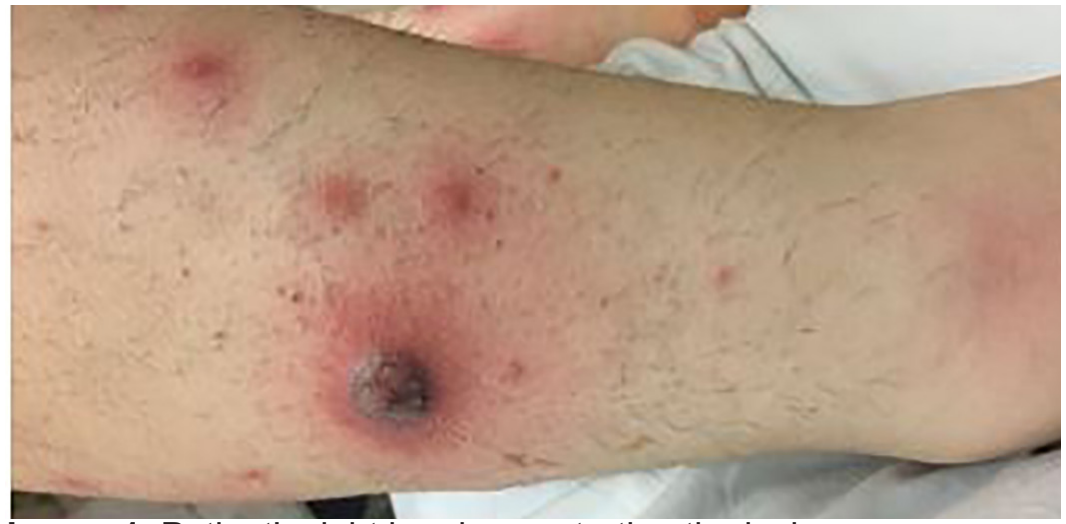
Patient’s right leg demonstrating the lesions.

**Image 2:**
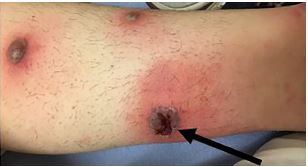
Patient’s left leg with site of incision and drainage marked by arrow.

The patient was a 33-year-old female with a history of untreated ulcerative colitis who presented to the emergency department for evaluation of painful leg lesions. The lesions had developed four days prior and she presented to an outside facility where incision and drainage was performed on one of the lesions. She was prescribed doxycycline on discharge. The lesions continued to spread and were associated with worsening pain. On exam she had multiple, tender violaceous nodules with surrounding erythema on the legs and one similar lesion on the right antecubital fossa at the site of a prior intravenous catheter from the outside facility (Images 1 and 2).

Laboratory results were notable for elevated inflammatory markers. A wound culture was negative. The patient was admitted for further workup and pain control following treatment with intravenous methylprednisolone. A biopsy later performed ultimately supported the suspected diagnosis of pyoderma gangrenosum.

## DISCUSSION

Pyoderma gangrenosum is a rare ulcerative skin condition frequently associated with systemic disease, most often inflammatory bowel disease.^1^ It is classically considered a “diagnosis of exclusion,” although a recent consensus statement proposes criteria to aid diagnosis (Table).^2^ A diagnosis of pyoderma gangrenosum is met with the major criterion and at least four of eight minor criteria with a sensitivity of 86% and a specificity of 90%.

This patient exhibited several classic features of pyoderma gangrenosum, including a history of inflammatory bowel disease, rapidly progressing painful ulcerative skin lesions with surrounding erythema, and evidence of pathergy (appearance of lesions at sites of trauma). Treatment of pyoderma gangrenosum is aimed at addressing the underlying disease process.^3^ Systemic corticosteroids are often required for successful treatment.^4^ Our patient was placed on a prednisone taper on discharge with improvement in symptoms and a plan for outpatient gastroenterology follow-up for ulcerative colitis control.

**CPC-EM Capsule**

What do we already know about this clinical entity?

*Pyoderma gangrenosum is a rare, painful ulcerative skin condition often associated with other systemic diseases typically treated with immunosuppressive medications.*

What is the major impact of the image(s)?

*This visual representation of pyoderma gangrenosum may assist with recognition for emergency providers.*

How might this improve emergency medicine practice?

*We review the presentation and management of pyoderma gangrenosum to aid providers in delivering effective and timely treatment.*

## Figures and Tables

**Table:** Diagnostic criteria for pyoderma gangrenosum.^2^

Major criterion: Biopsy demonstrating neutrophilic infiltrate at the ulcer’s edge
Minor criteria:
Infection excluded
Pathergy
History of inflammatory bowel disease or inflammatory arthritis
History of papule, pustule, or vesicle ulcerating within four days of appearing
Peripheral erythema, undermining border, and tenderness at ulceration site
Multiple ulcerations, at least one on an anterior lower leg
Cribriform or “wrinkled paper” scar at healed ulcer sites
Decrease in ulcer size within one month of initiating immunosuppressants

## References

[1] States V, O’Brien S, Rai JP (2020). Pyoderma gangrenosum in inflammatory bowel disease: a systematic review and meta-analysis. Dig Dis Sci.

[2] Maverakis E, Ma C, Shinkai K (2018). Diagnostic criteria of ulcerative pyoderma gangrenosum: a Delphi consensus of international experts. JAMA Dermatol.

[3] Miller J, Yentzer BA, Clark A (2010). Pyoderma gangrenosum: a review and update on new therapies. J Am Acad Dermatol.

[4] Partridge ACR, Bai JW, Rosen CF (2018). Effectiveness of systemic treatments for pyoderma gangrenosum: a systematic review of observational studies and clinical trials. Br J Dermatol.

